# Agreement between invasive and noninvasive measurement of tear film breakup time

**DOI:** 10.1038/s41598-024-54219-1

**Published:** 2024-02-15

**Authors:** Dorota H. Szczesna-Iskander, Clara Llorens-Quintana

**Affiliations:** 1 Department of Optics and Photonics, Faculty of Fundamental Problems of Technology, Wroclaw University of Science and Technology, Wybrzeze Wyspianskiego 27, 50-370 Wroclaw, Poland; 2 Department of Optometry and Vision, Faculty of Optics and Optometry, Complutense University of Madrid, Madrid, Spain

**Keywords:** Biomarkers, Diseases, Health care, Medical research

## Abstract

The agreement between fluorescein breakup time (FBUT) estimates and noninvasive breakup time (NIBUT) estimates automatically acquired using two videokeratoscopes was assessed. Small-cone (E300, Medmont) and large-bowl (K5M, OCULUS) Placido-ring–based videokeratoscopes were utilized for automated NIBUT estimation and fluorescein strip method was used for FBUT estimation. 33 subjects completed the study. Clear instructions were given regarding the blink before measurements. Bland–Altman analysis was used to test the agreement between tear film breakup time estimates and repeated measure analysis of variance was used to test the differences between measurement types. In comparison to FBUT, E300 NIBUT estimate was shorter (− 0.6 ± 2.6 s), whereas K5M NIBUT estimate was longer (3.3 ± 2.4 s). Limits of agreement for FBUT vs. E300 was 29.8 s, for FBUT vs. K5M 26.4 s, and 31.4 s for E300 vs. K5M. There were significant differences between tear film breakup times (*F* = 3.59, *df* = 2, *P* = 0.032). E300 and K5M NIBUT measurements have poor agreement with FBUT, even when blinking is precisely clarified. Agreement was better for shorted break up times than that for longer ones but in general NIBUT measurements are not interchangeable.

## Introduction

The fluorescein tear film breakup time (FBUT) measurement is a clinical test that is routinely used to assess the stability of the tear film despite its invasiveness. This test requires the application of fluorescein dye into the eye and the use of a slit lamp biomicroscope with a cobalt-blue filter to determine the time of appearance of the first dark spot on the tear film after a blink. Although this is a common clinical test, the Dry Eye Workshop Report (DEWS II) highlighted the benefits of using noninvasive techniques instead^1,2^. Accordingly, a number of instruments with the option to measure the noninvasive tear film breakup time (NIBUT) have become available, enabling eye-care practitioners to estimate NIBUT objectively and becoming a valid clinical biomarker for the assessment of tear film stability^3–10^.

An approach to estimate NIBUT is to look at the reflection of an illuminated grid pattern on the tear film surface and measure the elapsed time from a complete blink until the appearance of some distortion in the reflected pattern. In the past, this was evaluated subjectively, leading to errors and bias dependent on the observer’s interpretation of the temporal evolution of the projected pattern. Therefore, several manufacturers of Placido-ring–based corneal topographers have introduced modules for automated measurement of NIBUT. This enables eye-care practitioners to estimate NIBUT objectively.

While there are reasonable concerns about fluorescein destabilizing tear film during the measurement of FBUT, not all studies have found that NIBUT estimates are longer than those of FBUT^11^. The proposed dry eye diagnosis cut-off value for FBUT is 10 s when higher amounts of fluorescein are used^1,12^ and 5 s for micro-quantities^13^. However, it is important to note that FBUT result depends not only on the volume of fluorescein applied but also on other factors such as fluorescein concentration, method of instillation, time between instillation and measurement, instructions given to patient regarding blinking and method of observation including illumination, filter used, and width of the light slit^12^. Therefore, to standardize a cut-off value for FBUT, it would be necessary to take other variables into account, in addition to fluorescein quantity^14^. For NIBUT, DEWS II recommended to apply the 10-s cut-off value^1^. However, some studies suggest using other cut-off values for different Placido-ring–based instruments with automated software^3,9,15,16^. Comparative studies of various NIBUT measures have shown that although the results are correlated, they differ significantly and are not directly interchangeable^9,17,18^. Those differences may be attributed to the specific technical characteristics of each instrument such as the size of the bowl/cone, number and width of the Placido rings, as well as the approaches to image analysis^19^. In a previous study, tear film breakup time was shown to be affected by the unnaturally forced blink that patients may perform if not explicitly instructed prior to measurement^8^. Therefore, it is important to instruct patients before assessing tear film breakup time to ensure measurement conditions are as stable and equal as possible.

Given the discrepancies found between different estimators of tear film breakup time, the aim of this study was to assess the agreement between the standard FBUT estimates and those of automated NIBUT estimates measured by two videokeratoscopes in semi-controlled blinking conditions.

## Methods

### Subjects

The study followed the tenets of the Declaration of Helsinki and was approved by the Institutional Review Board of the Complutense University of Madrid. Each subject received both written and oral information about the aim and possible adverse effects of the study and signed a written informed consent. The exclusion criteria were history of ocular surgery, ocular infection or inflammation, any ocular systemic or autoimmune disease, age under 18 years, pregnancy or lactating as well as contact lens wear in last three days before the measurements. Compromised tear film quality or dry eye disease were accepted since the emphasis of the study was placed on within-subject tear film breakup time differences between methods of measurement regardless of tear film status. Based on the variation of FBUT and NIBUT estimates from K5M^11^, a sample size of 25 subjects provides 90% statistical power at a 5% significance level to detect a 1-s difference between measurements. The sample size requirement for correlation $(r\ge 0.500)$ being significantly different from the null correlation of zero at 80% power and 5% level of significance is 29 subjects^20^. Ultimately, 33 subjects were recruited for the study.

### Instrumentation

An RS1000 (Righton, Japan) slit lamp biomicroscope was used to assess FBUT. A drop of saline solution was placed on a fluorescein strip (Madhu Instruments Pvt. Ltd. New Delhi, India). After removing excess saline, the strip was gently applied to the temporal canthal lid margin area. Subjects were instructed to blink as naturally as possible several times to distribute fluorescein uniformly along the ocular surface and then they were asked to look straight ahead without blinking as long as they could. The slit lamp biomicroscope magnification was set at × 10; cobalt blue light and a Wratten 12 yellow filter (Eastman Kodak, Rochester, NY) were used to enhance observation of the tear film over the entire cornea. The time between a complete blink and the first appearance of the dark spot was recorded using a digital stopwatch.

Two Placido-ring-based videokeratoscopes were utilized to measure NIBUT: the E300 (Medmont Pty., Ltd., Melbourne, Australia), which is a small-cone videokeratoscope, and the Keratograph 5M (Oculus Optikgeräte, Wetzlar, Germany), which is a large-bowl videokeratoscope. The E300 projects 32 narrow rings, using RGB illumination (λ = 660/565/430 nm), and images of reflected Placido rings are recorded with the sampling frequency of 4 Hz, whereas the Keratograph 5M projects 22 wide rings using IR light illumination (λ = 880 nm), and images are recorded with a sampling frequency of 16 Hz. In both instruments image acquisition is triggered automatically when the camera is aligned and the subject blinks twice in a row. The measurements are automatically terminated when the subject blinks again or after reaching the maximum recording time, which was set in both instruments to 25 s. Both instruments incorporate software that allows objective and automated estimation of NIBUT but they use different proprietary image analysis approaches to do so.

### Study design

**Figure 1:**
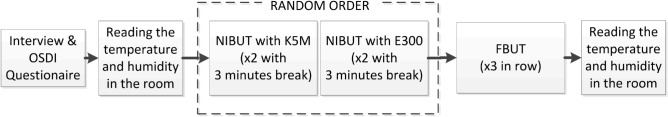
The measurement procedures and their order.

All measurements were performed by the same experienced clinician (CLQ) in the same room and on right eyes only. The measurements were taken between 9:00 AM and 2:00 PM. The humidity and temperature in the room were monitored. The measurement procedures are summarized in Fig. 1, the details were described in Szczesna-Iskander and llorens-Quintana^8^. All procedures were performed in a single day visit and took approximately 30 min per subject. Before any breakup time measurements, all subjects completed the Ocular Surface Disease Index (OSDI) and their medical history was reviewed. For breakup time measurements, subjects were asked to do two close-to-natural blinks to start the measurement. Therefore, careful explanation was given to each subject to ensure that this type of blink is well understood. The close-to-natural blink was explained to the subjects as a short involuntary blink, close to a quick blink normally performed subconsciously. The goal was to obtain a repeatable blink in each apparatus and keep the tear film distribution as close to natural conditions as possible.

NIBUT with both videokeratoscopes was measured before FBUT to avoid any interaction of the fluorescein with the measurement. The order of K5M and E300 was randomized to prevent any possible ordering effect. For NIBUT, two repeated measurements were taken with 3-min breaks between them to allow tear film recovery after prolonged eye opening. FBUT was measured at the end after another 3-min break, and three times in a row after single application of fluorescein as it is usually performed in the clinical practice. The lack of 3-min breaks between FBUT measurements was due to the fluorescein washout, which leads to reduced visibility of tear film break up, as well as because the subject could blink after the first black spot was seen, in contrast to NIBUT measurements, where the subject was asked to keep the eye open as long as possible until the maximum of 25 s that more likely created eye fatigue. All repeated measures were averaged for further analysis.

### Statistical analyses

The hypothesis of normal distribution of the tear film breakup time data was assessed by Shapiro–Wilk (SW) test with the level of significance set at 0.05. Upon rejection, the distribution of the data was evaluated by kernel density estimator with a positive support and a Gaussian kernel of standard width of h = 1.06σN^(−1/5)^. This was followed by appropriately transforming the data^21^ and testing again the hypothesis of normal distribution. The agreement between FBUT and NIBUT estimates was assessed by means of Bland–Altman plots where the mean difference and the limits of agreement are presented. The limits of agreement are calculated as the average difference ± 1.96 times the SD of the difference measure. Repeated measure analysis of variance (ANOVA) was performed for testing the differences between measurement types. For post-hoc analysis the t-test for dependent variables was performed. Level of significance was set to 0.05 for all tests except for the post-hoc analysis where it was 0.017 to allow compensation for multiple comparisons—Bonferroni correction. Calculations and graphs were performed using MATLAB (MathWorks, Natick, MA). Statistical correlation between tear film breakup time estimators was assessed using Pearson’s correlation coefficient. Partial correlation was used to assess the confounding effect of OSDI, as well as the changes in room’s humidity and temperature during the visit.

## Results

**Figure 2:**
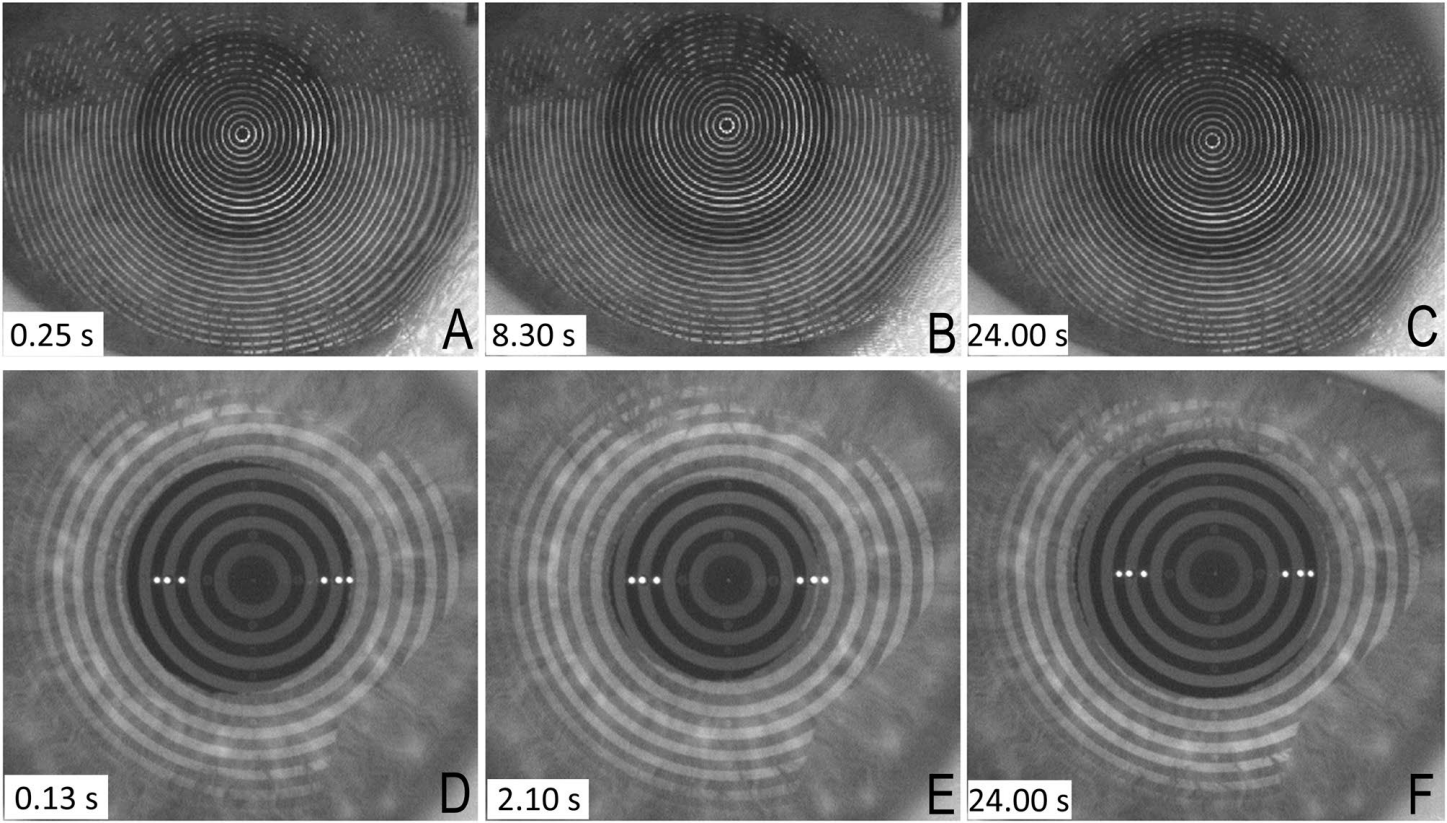
Examples of recorded frames with the two considered videokeratoscopes for two different subjects. Upper row—E300 (Medmont): (**A**) the first recorded frame after blink, (**B**) frame recorded at 8.30 s when the automatic software identified NIBUT and (**C**) 24.00 s after blink (end of the sequence). Lower row—K5M (Oculus): (**D**) the first recorded frame after blink, (**E**) frame recorded at 2.10 s when the automated software identified NIBUT and (**F**) 24.00 s after blink (end of the sequence).

This cross-sectional study was performed with thirty-three subjects aged between 23 and 53 years. The characteristics of the cohort as well as the environment conditions are summarized in Table 1. In nine subjects the OSDI result was above 13 while FBUT was below 10 s, hence meeting the diagnostic criteria for dry eye according to the DEWS II Report. Figure 2 presents illustrative frames from two sequences recorded with E300 (Fig. 2A–C) and K5M (Fig. 2D–F) videokeratoscopes. Automated NIBUT was indicated at 8.3 s (B) and at 2.1 s (E) after blink, respectively in E300 and K5M. The FBUT averaged result was 4.0 s and 2.4 s, for the subject from E300 example and for that from K5M example, respectively.

**Table 1:** Subjects and environment characteristics.

Characteristic	Mean ± SD; [range]
Age (years)	28 ± 6; [23/53]
OSDI	12.39 ± 8.51; [0.0/34.1]
Temperature in the room (°C)	23.91 ± 1.87; [19.9/25.9]
Relative humidity in the room (%)	32.85 ± 10.06; [16.6/48.3]

*OSDI* Ocular Surface Disease Index, *SD* standard deviation.

**Figure 3:**
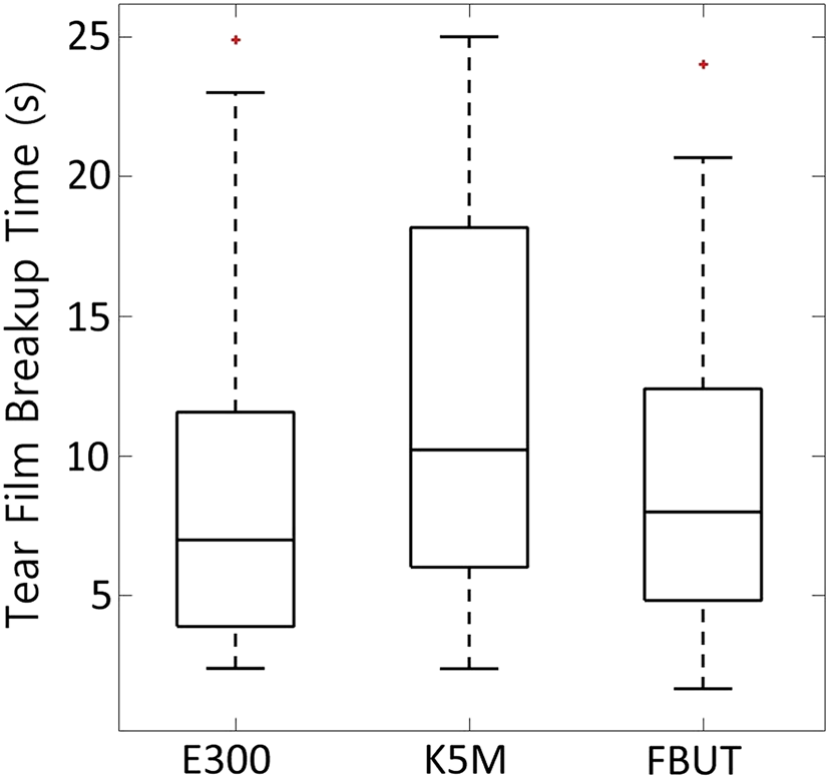
Boxplots of tear film breakup times measurements, obtained from E300, K5M, and FBUT. Red crosses denote outliers calculated using the method of Tukey fences with coefficient of 1.5.

Normality of the data was first rejected. The obtained results for E300, K5M and FBUT were as follows (SW test): P = 0.003, P = 0.017, and P = 0.007, respectively. Kernel density estimators revealed positive skew for all types of measurements with estimates of it being ${\upbeta }_{1}$= 1.122, ${\upbeta }_{1}$ = 0.380, and ${\upbeta }_{1}$ = 0.949 for E300, K5M and FBUT, respectively). Logarithmic transformation of the data was applied. At further testing, normality of the data was not rejected; the results for E300, K5M and FBUT were as follows (SW test): P = 0.235, P = 0.085, and P = 0.867, respectively. The repeated measures ANOVA showed significant differences between tear film breakup time ($F=3.28,df=2, P=0.042$). Figure 3 presents the boxplot of group results separately for each type of measure. One-tailed paired samples t-test revealed no statistically significant differences when FBUT was compared to NIBUT estimated by E300 videokeratoscope $(P=0.319)$ but there was statistically significant differences between FBUT and NIBUT estimated by K5M videokeratoscope $(P=0.004)$. Also, statistically significant difference was found between NIBUT estimates from both instruments $(P<0.001)$ where shorter NIBUT was measured by E300.

Regarding correlations between the measurements, a significant correlation was found between FBUT and NIBUT from E300 $$(r=0.488, P=0.004)$$ and between FBUT and NIBUT from K5M $$(r=0.552, P<0.001)$$. Fisher test for correlation coefficient resulted in statistically insignificant ($$P>0.05)$$ differences between the considered correlations and their partial counterparts with OSDI and the differences between temperature and humidity being the control variables, indicating that they do not confound the correlation results.

**Figure 4:**
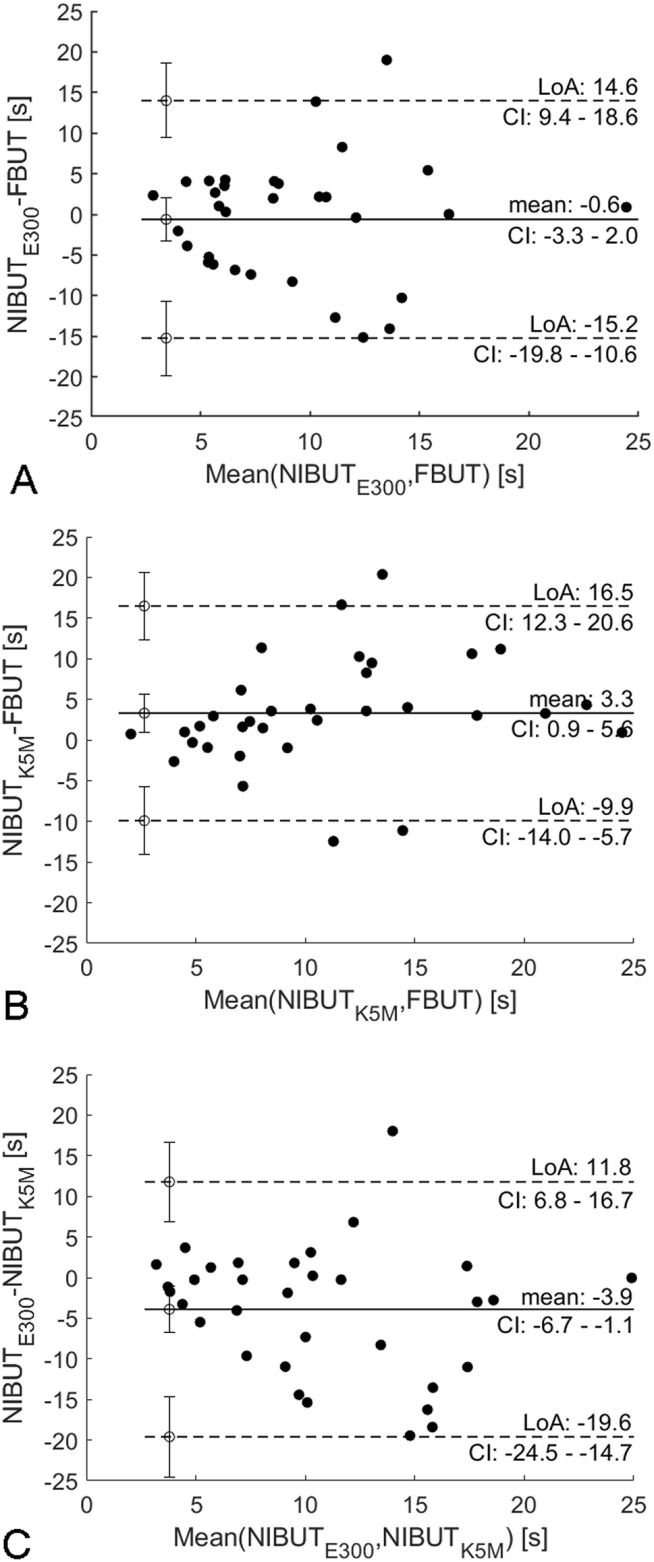
Bland–Altman plots showing the agreement between objective noninvasive break up time (NIBUT) and fluorescein break up time (FBUT) for E300 (**A**) and K5M (**B**) videokeratoscopes, and the agreement between noninvasive break up time (NIBUT) between E300 and K5M videokeratoscopes (**C**). Solid lines represent the mean difference and dashed lines the limits of agreement. The error bars show the confidence intervals (CI) for the limits of agreement (LoA) and the mean.

**Figure 5:**
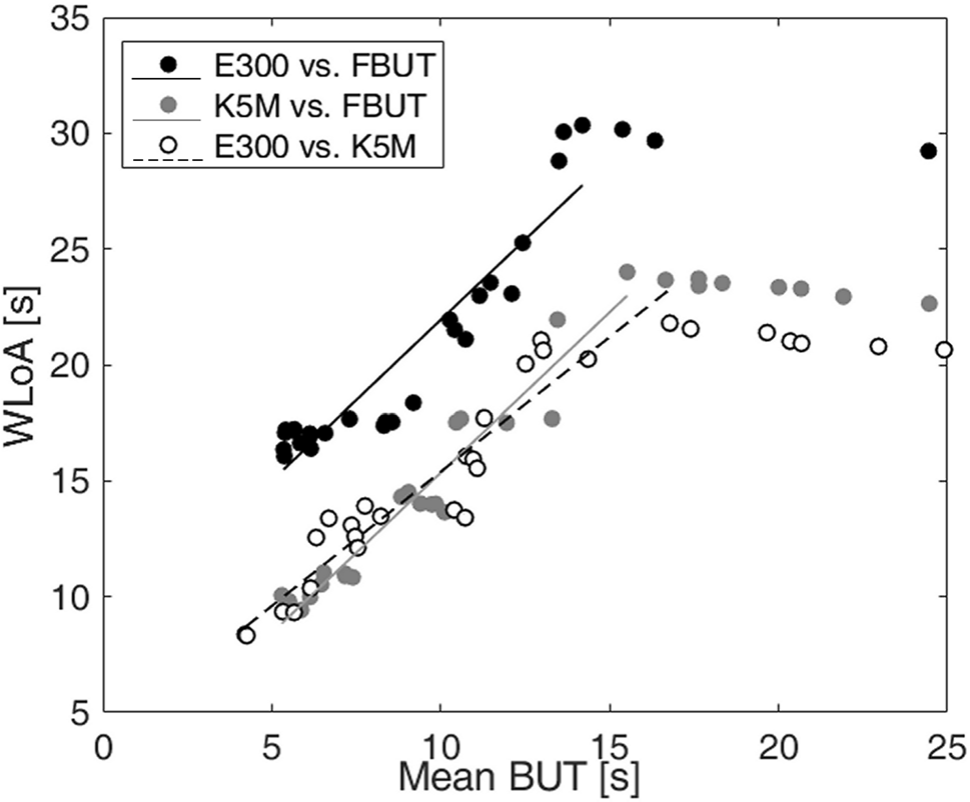
The width of the limits of agreement (WLoA) as a function of mean tear film breakup time (BUT) and the best-fit line up to value at which the WLoA is maximum for: NIBUT from E300 vs. FBUT—black circles and black line, NIBUT from K5M vs. FBUT—gray circles and gray line, and NIBUT from E300 vs. K5M—white circles and dashed line.

Figure 4 shows the agreement between NIBUT and FBUT (A and B) as well as between the two videokeratoscopes (C) by means of Bland–Altman plots. Compared to FBUT, E300 gave shorter breakup times (mean difference ± one standard deviation): − 0.6 ± 2.6 s, whereas K5M gave longer breakup times: 3.3 ± 2.4 s. On average, E300 gave shorter breakup times compared to 5KM: − 3.9 ± 2.8 s. All Bland–Altman plots show lower agreement between methods of measurement (FBUT vs. NIBUT) and instruments (E300 vs. K5M), particularly for longer tear film breakup times. Therefore, the width of the limits of agreement (WLoA), defined as the difference between the upper and lower limit of agreement, was examined for different mean breakup times starting from just below 5 s (the strict cut-off value for dry eye diagnosis) to 25 s (the maximum time of measurement). Figure 5 presents the WLoA as a function of mean tear film breakup time. All plots show that for mean BUT values of up to about 15 s WLoA increases and above those values it plateaus. The best-fit line (*f(x)* = *bx* + *a*) to WLoA for mean BUT values up to that at which the WLoA is maximum is (slope *b*, Pearson correlation coefficient *R*, and the *P*-value): *b* = 1.386, *R* = 0.937, and *P *$\ll$ 0.001 for E300 vs. FBUT; *b* = 1.383, *R* = 0.963, and *P* $\ll$ 0.001 for K5M vs. FBUT and *b* = 1.160, *R* = 0.950, and *P* $\ll$ 0.001 for E300 vs. K5M.

Since the 10-s threshold is a commonly used cut-off value in dry eye diagnosis, it was chosen for comparing the limits of agreement below and above it for the three pairs of instruments/methods (Table 2). The Bland Altman plots are shown in the Supplementary material. Each is a merger of two separate Bland–Altman plots for mean tear film breakup times below and above the 10-s threshold.

**Table 2:** The mean and the limits of agreement (LoA) with confidence intervals (CI) calculated separately for mean tear film breakup time for: NIBUT from E300 vs. FBUT, NIBUT form K5M vs. FBUT and NIBUT from E300 vs. K5M, respectively, below and above 10 s.

	Upper LoA [CI]		Mean [CI]		LoA lower [CI]	
	< 10s	≥ 10s	< 10s	≥ 10s	< 10s	≥ 10s
E300 vs. FBUT	8.2 [4.4, 12]	20.8 [9.6, 31.9]	− 0.9 [− 3.2, 1.2]	− 0.1 [− 6.5, 6.4]	− 10.2 [− 13.9, − 6.4]	− 20.9 [− 32.0, − 9.8]
K5M vs. FBUT	8.3 [5.2, 11.4]	19.5 [12.7, 26.2]	1.3 [− 0.5, 3.0]	5.7 [1.8, 9.6]	− 5.7 [− 8.8, − 2.6]	− 8.0 [− 14.7, − 1.3]
E300 vs. K5M	5.2 [2.0, 8.3]	5.2 [0.1, 10.4]	− 1.5 [− 3.4, 0.3]	− 6.1 [− 9.1, − 3.2]	− 8.3 [− 11.5, − 5.1]	− 17.4 [− 22.6, − 12.3]

## Discussion

This study investigated the differences between tear film breakup time estimates provided by the fluorescein test and given by the two videokeratoscopes. FBUT was not significantly different from NIBUT when assessed with E300 but it was significantly shorter compared to K5M NIBUT. The relationship between FBUT and NIBUT has been previously reported in other studies^3,10,22,23^, but there is no consensus within them. There are several factors that may contribute to this lack of consistency among all studies. FBUT is influenced by the amount of fluorescein instilled^12^ and the method used for instillation^10^, and they were different in those studies. In addition, it was shown that FBUT results have poor repeatability and reproducibility increasing the variance of the estimates^24,25^. Contrarily, the NIBUT measurements have the potential to be more repeatable and reproducible because of their objective character when evaluated in an automated fashion. In general, it is difficult to assess the repeatability of measuring some quantity that is dynamic in nature and depends on many external conditions. Nevertheless, in a noninvasive measurement of tear film breakup time there are less factors that may influence the result than in the case of FBUT measurement. Taking aside those factors the present study agrees with the results obtained by other authors where FBUT was shorter than NIBUT from K5M pointing to an argument that fluorescein instillation may reduce tear film stability^10^. However, there are studies that show contrary results reporting that the NIBUT can be significantly shorter than the FBUT, as in the work of Hong et al.^11^, who used Oculus videokeratoscopy for NIBUT measurement.

It has been argued that different NIBUT results obtained from different videokeratoscopes are not comparable due to the different technical characteristics and different software used to analyze the images^9,14,19^. This is also supported by the results obtained in this study, where NIBUT estimates between E300 and K5M showed to be statistically significantly different. In the study by Lim et al.^9^, clinically and statistically significantly longer NIBUT was obtained from E300 compared to K5M, while this was opposite in this study, where E300 gave statistically and clinically significantly shorter NIBUT. The difference between these two studies, apart from putting attention to blink consistency in both instruments, was that in the study of Lim et al.^9^ measurements were carried out in an air-conditioned room, whereas in this study the room had no air-conditioning. The design of both studies put attention to minimize the eye fatigue. Lim et al.^9^ partially explained the longer NIBUT obtained by K5M by the higher exposure of the ocular surface to the ambient conditions in the bowl of K5M, whereas the cone of E300 is positioned much closer to the eye and it may be a physical barrier for the airflow that promotes aqueous tear evaporation and tear film destabilization^26^. A possible reason for the opposite results may be the effect of higher airflow velocity in the air-conditioned room compared to the conditions in the room where the measurements of this study were carried out.

The Bland–Altman analysis revealed that the agreement between measurement methods is better for shorted tear film breakup times (see Fig. 4). The width of the limits of agreement (WLoA) shown in Fig. 5 increases quasi-monotonically indicating the dependence of agreement between instrument and methods of measurements on the tear film breakup time. Nevertheless, these plots show that for less stable tear film there is higher agreement between invasive and noninvasive measurements as well as between K5M and E300. Considering that many studies use 10 s for the cut-off value for dry eye disease, as suggested by the DEWS II report, it can be concluded that agreement is better for measurements in dry eye suspects and patients for whom assessment of tear film quality is more important than for healthy individuals. Lan et al.^22^ using K5M also indicated that the longer the tear film breakup time, the greater the discrepancy between NIBUT and FTBUT.

It is speculated that short tear film breakup times are associated with more abrupt changes in tear film surface characteristics that are easily detected both by software (NIBUT) or the observer (FBUT) compared to long tear film breakup times where tear film changes are slower and less obvious.

The large limits of agreement observed here and by other researchers^10^ suggest that Placido-ring–based NIBUT measures are not equivalent substitutes for FBUT. However, this conclusion has been largely drawn by measurements on a mostly healthy cohort of subjects. The fact that the agreement between FBUT and NIBUT is higher with a less stable tear film suggests that NIBUT testing may perform better in a dry eye cohort of subjects. However, Abdelfattah et al.^27^ concluded the inability of the automated measures by K5M to differentiate between the control and ocular surface disease groups in contrast to FBUT. The challenge is to establish a cut-off value for NIBUT assessment that, according to previous studies, should be different for different instruments^3,9,15,16^. Another reason why there is a poor agreement between Placido-ring–based NIBUT estimates and those of FBUT is that the two methods may measure different phenomena. The fluorescein is sensitive to the change in local tear film volume whereas the pattern reflection shows disturbances on the tear film surface. E300 and K5M seem to overcome the problem of detecting local disturbance that does not develop in time from a foreign matter or lipid clumps, for example, but unjustifiably short estimates of tear film breakup time also occur. It is worth emphasizing that the algorithms from K5M and E300 have the potential to detect tear film breakup in the recordings where an observer does not notice irregularity or any other disturbance in the reflected rings, and such results were occasionally found to agree with their FBUT equivalents.

The limitation of this study that could not be overcome is the order of the measurements taken, keeping the fluorescein test always at the end of the visit. Nevertheless, this measurement did not give the shortest average result, which otherwise could have been expected due to eye fatigue. Another limitation, that could be also a possible reason of disagreement observed in most of similar studies, is that the FBUT was averaged from measurements performed in row after single fluorescein instillation, whereas NIBUT estimates were averaged from separate measurements separated by 3 min. The subjectivity of the FBUT assessment should also be considered as limitation because the assessment from one observer was compared to automatic objective evaluation.

In conclusion, although the blink type was semi-controlled, this study confirmed that the estimates of tear film breakup time from K5M and E300 cannot be used interchangeably. Also, it appears that noninvasive measurement of tear film breakup time is not a substitute for FBUT as yet. The cut-off values for NIBUT should be revised and verified since the values adopted for FBUT are not transferrable. Also, the origin of the Placido rings distortion pattern requires better understanding. The NIBUT estimates from E300 gave better agreement with FBUT than those of K5M, but it also showed weaker correlation with the FBUT, which is considered a standard due to its better correlation with other clinical measures of anterior eye health^28,29^. Hence, the results of NIBUT measurements from different studies using different instruments should be compared with caution. It has been established that standardization of the FBUT test is impossible^14^ because there are many variables that may be different between studies and measurements. For NIBUT, however, such standardization could be envisaged. Efforts should be made to arrive at such standard NIBUT measurement procedure in the future.

### Supplementary Information

Supplementary Figure 1.

## Data Availability

Raw data are available with the first author (DSI) and can be shared upon reasonable request.
