# Supplementary material for: Agreement between invasive and noninvasive measurement of tear film breakup time
Source: Sci Rep. 2024 Feb 15;14:3852. doi: 10.1038/s41598-024-54219-1 (PMC10869834; doi:10.1038/s41598-024-54219-1)
Supplement: Supplementary file 1 — Supplementary Figure 1. [file 41598_2024_54219_MOESM1_ESM.docx]

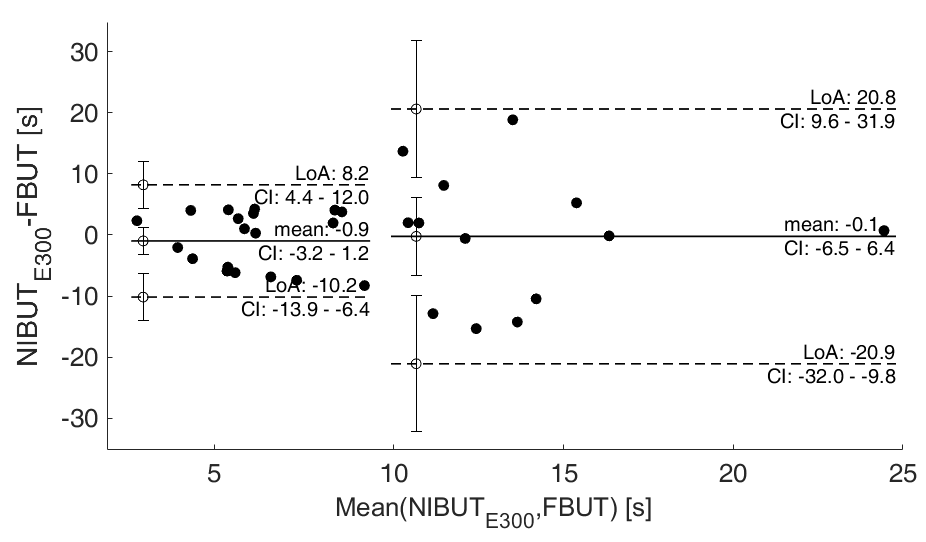
A


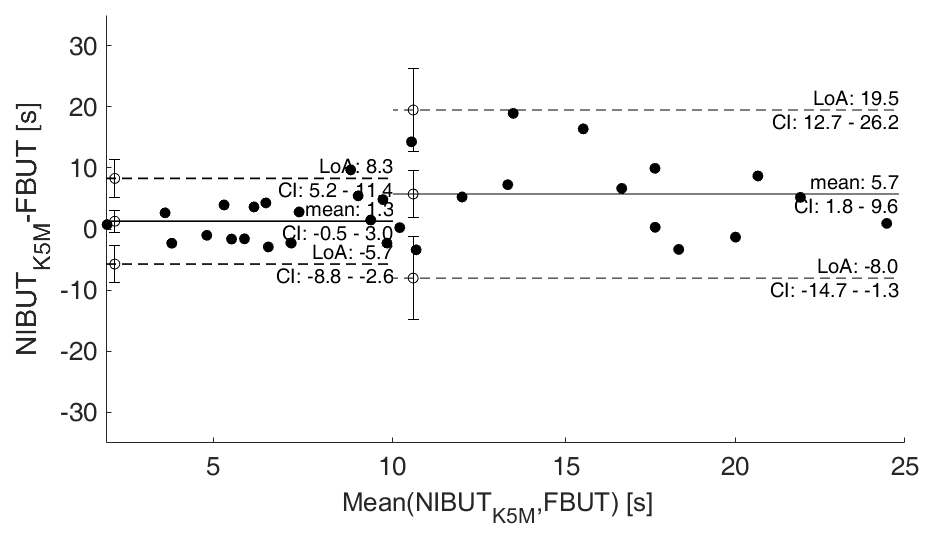
B


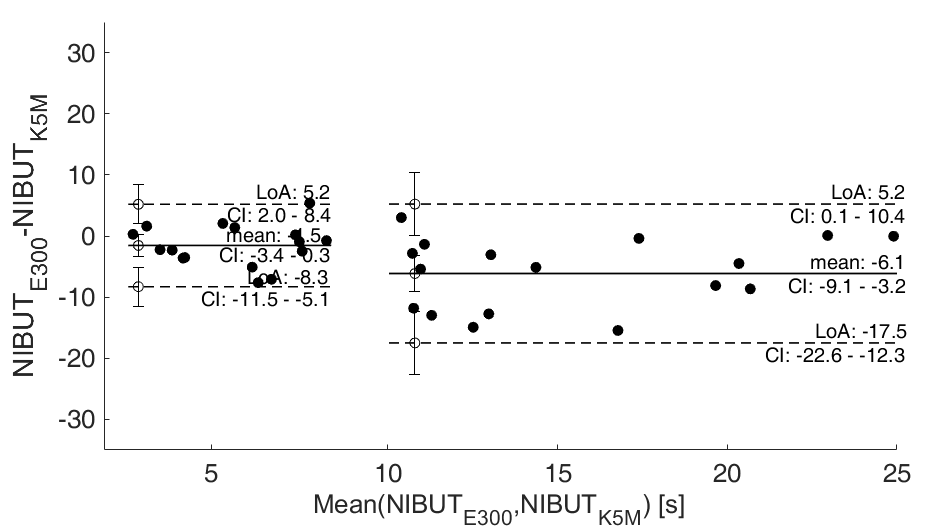
C

Figure. The merger of two separate Bland-Altman plots for mean tear film breakup times showing the agreement between objective noninvasive break up time (NIBUT) and fluorescein break up time (FBUT) estimates for E300 (**A**) and K5M (**B**) videokeratoscopes, and the agreement between noninvasive break up time (NIBUT) between E300 (Medmont Pty., Ltd., Melbourne, Australia) and K5M (Oculus Optikgeräte, Wetzlar, Germany) videokeratoscopes (**C**) below and above the 10 seconds. Solid horizontal lines represent the mean difference and dashed lines the limits of agreement. The error bars show the confidence intervals (CI) for the limits of agreement (LoA) and the mean.
